# Persistent headache after first-ever ischemic stroke: clinical characteristics and factors associated with its development

**DOI:** 10.1186/s10194-022-01479-9

**Published:** 2022-08-17

**Authors:** Elena R. Lebedeva, Anton V. Ushenin, Natalia M. Gurary, Denis V. Gilev, Nadezda V. Kislyak, Jes Olesen

**Affiliations:** 1 Department of Neurology, the Ural State Medical University, Repina 3, Yekaterinburg, 620028 Russia; 2 International Headache Center “Europe-Asia”, Yekaterinburg, Russia; 3 Medical Union “New Hospital”, Yekaterinburg, Russia; 4 Department of Economics, the Ural Federal University, Yekaterinburg, Russia; 5 Department of Neurology, Danish Headache Center, Rigshospitalet-Glostrup, University of Copenhagen, Copenhagen, Denmark

**Keywords:** Headache, Persistent headache, Stroke, Post-stroke pain, Medication overuse headache, International classification of headache disorders

## Abstract

**Background:**

It is poorly described how often headache attributed to stroke continues for more than 3 months, i.e. fulfils the criteria for persistent headache attributed to ischemic stroke. Our aims were: 1) to determine the incidence of persistent headache attributed to past first-ever ischemic stroke (International headache society categories 6.1.1.2); 2) to describe their characteristics and acute treatment; 3) to analyse the prevalence of medication overuse headache in patients with persistent headache after stroke; 4) to evaluate factors associated with the development of persistent headache after stroke.

**Methods:**

The study population consisted of 550 patients (mean age 63.1, 54% males) with first-ever ischemic stroke, among them 529 patients were followed up at least three months after stroke. Standardized semi-structured interview forms were used to evaluate these headaches during professional face-to-face interviews at stroke onset and telephone interviews at 3 months.

**Results:**

At three months, 61 patients (30 women and 31 men, the mean age 60.0) of 529 (11.5%) follow-up patients had a headache after stroke: 34 had a new type of headache, 21 had a headache with altered characteristics and 6 patients had a headache without any changes. Therefore 55 (10.4%) patients had a persistent headache attributed to ischemic stroke. Their clinical features included: less severity of accompanying symptoms, slowly decreasing frequency and development of medication overuse headache in one-third of the patients. The following factors were associated with these headaches: lack of sleep (29.1%, *p* = 0.009; OR 2.3; 95% CI 1.2–4.3), infarct in cerebellum (18.2%, *p* = 0.003; OR 3.0; 95% CI 1.4–6.6), stroke of undetermined etiology (50.9%, *p* = 0.003; OR 2.3; 95% CI 1.3–4.1), less than 8 points by NIHSS score (90.9%, *p* = 0.007; OR 3.4; 95% CI 1.4–8.6) and low prevalence of large-artery atherosclerosis (12.7%, *p* = 0.006; OR 0.3; 95% CI 0.2–0.80).

**Conclusion:**

Persistent headache attributed to ischemic stroke is not rare and frequently leads to medication overuse. The problem is often neglected because of other serious consequences of stroke but actually, it has a considerable impact on quality of life. It should be a focus of interest in the follow-up of stroke patients.

## Introduction

In the International Classification of Headache Disorders, 3^rd^ edition (ICHD-3) distinction is made between acute headache attributed to ischemic stroke and persistent headache attributed to ischemic stroke. The latter is defined as an acute headache attributed to ischemic stroke that persists for 3 months or more. We and others have extensively studied the acute type, but persistent headache attributed to ischemic stroke has received much less attention and nobody has studied this headache strictly according to ICHD-3 diagnostic criteria or the modified improved criteria proposed by us [1]. Only headaches after a first-ever ischemic stroke should be taken into account, otherwise, causality is difficult to establish. It is also important to document that reported headaches are a continuation of a headache attributed to ischemic stroke and not just a pre-existing headache that continued. But these requirements have generally not been fulfilled by previous studies. We consider persistent headache attributed to ischemic stroke to be an important entity because it may considerably reduce the quality of life of stroke victims. Its diagnosis and treatment seem to be overshadowed by other stroke sequelae that may in fact be less debilitating.

We previously suggested diagnostic criteria for acute headache attributed to ischemic stroke that distinguish between a new type of headache and a previous headache with altered characteristics [2]. Both types are caused by the stroke and both can lead to persistent headache after stroke. Factors associated with the development of persistent headache after stroke have not been studied before*.* The aims of our study were the following: 1) to determine the incidence of persistent headache attributed to past first-ever ischemic stroke (International headache society categories 6.1.1.2); 2) to describe their characteristics and acute treatment; 3) to analyse the prevalence of medication overuse headache in patients with persistent headache after stroke; 4) to evaluate factors associated with the development of persistent headache after stroke.

## Material and methods

### Patients

Patients were consecutively recruited from the stroke unit of city hospital “New Hospital” in Yekaterinburg, Russia. They had first-ever ischemic stroke and were included if they were 18 years and older, had a new infarction on magnetic resonance imaging (MRI) with diffusion-weighted imaging (DWI), agreed to be interviewed and followed up not less than three months after acute headache attributed to stroke. Exclusion criteria were a history of previous stroke/transient ischemic attacks, intracranial/intracerebral haemorrhage, unruptured cerebral artery aneurysm, brain tumour, any brain surgery, traumatic brain injury, multiple sclerosis, epilepsy, encephalitis, meningitis and other severe neurological or somatic diseases that can provoke secondary headache disorders. A patient should be able to give a clear description of previous and current headaches.

We recruited 550 patients with first-ever ischemic stroke out of 2995 patients with ischemic stroke who met the above-described inclusion and exclusion criteria and agreed to participate in the study. All data were collected prospectively. The present study is a continuation of three previous studies about headache in first-ever ischemic stroke which described the prevalence of sentinel headache, headache at onset of first-ever ischemic stroke, their diagnostic criteria and associated factors [2–4]. A full description of the inclusion procedure has been published in the previous articles. The present study focuses on patients with persistent headache after stroke, their diagnostic criteria and risk factors for these headaches.

### Evaluation

The patients were assessed on the day of stroke and at last 3 months later, with regard to headache attributed to stroke. According to our previous study [3], these headaches included: 1) a new type of headache; 2) headache with altered characteristics. Both occur within 24 h of stroke onset.

Standardized semi-structured interview forms were used to evaluate headaches during face-to-face interviews at stroke onset in 550 patients with first-ever ischemic stroke and telephone interviews at 3 months in 529 follow-up patients after stroke. The first interview was done by two trained neurologists. A third neurologist interviewed patients by telephone at 3 months after stroke. He did not evaluate the patients previously and was blinded to the presence of headache at stroke onset. Therefore, he interviewed all patients who agreed to be follow-up including patients without acute headache attributed to stroke. Patients were asked whether they perceived any headache or not, what are characteristics of these headaches and whether the headache had changed. If the headache disappeared, the date of disappearance was recorded.

We evaluated the following characteristics of headache at onset of stroke and three months after that: 1) the number of attacks per week within 1^st^ month and at 1^st^, 2^nd^ and 3^rd^ months after stroke; 2) intensity of headache (mild, moderate, severe) during 1^st^, 2^nd^ and 3^rd^ months after stroke; 3) quality (pulsating, pressing, other); 4) location and side of headache; 5) aggravation by routine physical activity; 6) accompanying symptoms (nausea, vomiting, photo- and phonophobia); 7) the presence of aura and its symptoms (visual, sensory, speech, motor, other); 8) use of drugs for pain relief, preventive treatment of headache and stroke prevention/other drugs, kind and name of a drug, frequency, effect.

Characteristics of headache at stroke onset were compared to headaches at three months. A new type of headache and headache with and without altered characteristics were analysed separately. Type of headache (migraine-like, tension-type-like, etc.) was recorded.

The prevalence of factors possibly associated with persistent headache after stroke was compared to the prevalence in patients without headaches. These factors included: mean age, sex, lack of sleep, a history of hypertension, diabetes, atrial fibrillation, angina, myocardial infarction, peripheral artery disease, large-artery atherosclerosis ( ≥ 50% stenosis or occlusion of arteries on the neck which were verified during triplex ultrasonography or CT-angiography), smoking, alcohol consumption, body mass index > 25, family history of stroke, low physical activity (less than 30 minutes of physical exercises 1 time per week), NIHSS score, type of stroke (TOAST classification), localization and size of the infarct***.***

### Definitions and diagnostic criteria

Acute headache attributed to stroke was determined using our validated proposed diagnostic criteria [2]. Persistent headache after a first-ever ischemic stroke was defined according to ICHD-3 as an acute headache attributed to ischemic stroke that persisted for 3 months [1]. A new type of headache at stroke onset was defined as a headache which arose for the first time within 24 h after stroke onset. If patients had a pre-existing headache but the headache at stroke onset had changed in characteristics, these headaches were also attributed to stroke. If they had unaltered characteristics, they were not attributed to stroke. If a new type of headache or a headache with altered characteristics had a phenotype of migraine or tension-type headache they were named migraine-like and tension-type-like according to the general rules of ICHD-3.

Medication overuse headache was diagnosed based on the self-reported use of simple analgesics for pain relief as ≥ 15 days per month or triptans and/or combine analgesics ≥ 10 days/month for > 3 months [1].

Stroke location was defined as anterior (medium cerebral artery and anterior cerebral artery), posterior (posterior cerebral artery, cerebellum and brainstem), or subcortical (thalamus, internal capsule lesions).

### Statistical analysis

Data were analyzed with Stata 14.0 software (StataCorp LP, College Station, TX, USA) and Microsoft Excel (2014). Pearson’s 2 test or Fisher’s exact test was conducted for comparison of categorical variables depending on group sizes. T-test and Wilcoxon rank-sum were applied for continuous variables. Odds ratio (OR) was used to estimate associated factors. Two-tailed probability (*p*) values < 0.05 were considered significant.

When quantitative indicators were evaluated for compliance with a normal distribution, we used the Shapiro–Wilk test (when the number studied was less than 50) or the Kolmogorov–Smirnov test (when the number of investigated was more than 50). In the Chi-square calculation, if the expected number in at least one cell was less than 10, we calculated the χ2 with Yates’s correction.

Multivariate logistic regression analysis was performed to identify independent factors. Each covariate was evaluated individually; those meeting the significance level of *p* < 0.05 and OR > 1 were then included in multivariate models to identify their independent contributions after adjusting for the presence of all other variables. These factors were analyzed in participants with a headache at stroke onset compared to participants without a headache. All analyses were performed by two statisticians (DVG, NVK).

## Results

Eighty-two patients (42 women and 40 men) of 550 patients (14,9%) had an acute headache at a first-ever ischemic stroke. 46 had a new type of headache, 30 had a previous headache with altered characteristics and 6 patients had a previous headache without any changes. At three months follow-ups, 61 patients (30 women and 31 men, the mean age 60.0) of 529 follow-up patients (11.5%) had a persistent headache after stroke. Among them 34 had a new type of headache, 21 had a headache with altered characteristics and 6 patients had a headache without any changes (Table 1). Therefore 76 patients (13.8% of 550) patients had an acute headache attributed to first-ever ischemic stroke and 55 patients (10.4% of 529) patients had a persistent headache attributed to stroke.

**Table 1:** Types of headaches which persisted for ≥ 3 months after first-ever ischemic stroke among 529 follow-up patients

Type of headache	Number of patients
**Previous headache without changes**	6 (1.1%)
Migraine without aura	3 (0.6%)
Migraine with aura	0 (0%)
Tension-type headache	3 (0.6%)
Cluster headache	0 (0%)
**Previous headache with changes of characteristics**	21 (4.0%)
Migraine-like headache	5 (0.9%)
Tension type-like of headache	16 (3.0%)
Cluster-like headache	0 (0%)
**New type of headache**	34 (6.4%)
Migraine-like headache	20 (3.8%)
Tension-type-like headache	9 (1.7%)
Cluster-like headache	0 (0%)
Thunderclap-like headache	5 (0.9%)

The time window of development and disappearance of acute headache attributed to ischemic stroke varied for different types of headache. All new types of headache (*n* = 46) developed simultaneously with the onset of stroke. They disappeared within 24 h after stroke onset in 25 (54.3%) and continued for more than 24 h in 21 patients (45.7%). Previous headaches with altered characteristics were observed in 14 patients. They developed at stroke onset, and in 16 patients they developed during the 24 h after stroke onset.

We analysed the change of characteristics of headache at three months (intensity, accompanying symptoms, duration, location) and the use of drugs for pain relief compared to the onset of first-ever ischemic stroke (Table 2). The intensity of persistent new type of headache was almost unchanged. Persistent headache with altered characteristics became less severe (56.7 and 42.9%). Aggravation by routine physical activity and duration of headache attacks did not change in the two groups. The frequency of accompanying symptoms (photo-, phonophobia, vomiting) became significantly less in three months compared to the onset of stroke in both groups. The presence of hemicrania also decreased significantly, especially in persistent new type of headache at three months. Most persistent new type of headache became bilateral (79.4%). The side of infarction and side of persistent headache were not correlated.

**Table 2:** Changes in characteristics of persistent headaches during three months compare to initial characteristics at onset of first-ever ischemic stroke

Characteristics of headache	New type of headache		P, OR, 95% CI	Headache with altered characteristics		P, OR, 95% CI
	**Headache at the onset of stroke (*n* = 46)**	**Headache in three months after stroke (*n* = 34)**		**Headache at the onset of stroke (*n* = 30)**	**Headache in three months after stroke (*n* = 21)**	
**Frequency of headache per month**						
** First month**						
1–7 days	n/a^a^	7 (20.6%)		n/a	4 (19.0%)	
8–14 days	n/a	14 (41.2%)		n/a	9 (42.9%)	
≥ 15 days	n/a	13 (38.2%)		n/a	8 (38.1%)	
** Second month**						
1–7 days	n/a	11 (32.4%)		n/a	5 (23.8%)	
8–14 days	n/a	13 (38.2%)		n/a	8 (38.1%)	
≥ 15 days	n/a	10 (29.4%)		n/a	8 (38.1%)	
** Third month**						
1–7 days	n/a	13 (38.2%)		n/a	6 (28.6%)	
8–14 days	n/a	12 (35.3%)		n/a	7 (33.1%)	
≥ 15 days	n/a	9 (26.5%)		n/a	8 (38.1%)	
** Intensity**						
Mild	1 (2.2%)	0 (0%)	0.4	2 (6.7%)	0 (0%)	0.2
Moderate	14 (30.4%)	13 (38.2%)	0.5	11 (36.7%)	12 (57.1%)	0.1
Severe	31 (67.4%)	21 (61.8%)	0.6	17 (56.7%)	9 (42.9%)	0.3
** Duration**						
< 1 h	4 (8.7%)	0 (0%)	0.08	0 (0%)	0 (0%)	-
1–4 h	1 (2.2%)	0 (0%)	0.4	0 (0%)	0 (0%)	-
5–23 h	17 (37.0%)	13 (38.2%)	0.9	13 (43.3%)	9 (42.9%)	0.9
1–3 days	24 (52.2%)	21 (61.8%)	0.4	17 (56.7%)	12 (57.1%)	0.9
Aggravation by routine physical activity	31 (67.4%)	27 (79.4%)	0.2	24 (80.0%)	15 (71.4%)	0.5
** Accompanying symptoms**						
Nausea	22 (47.8%)	18 (52.9%)	0.7	9 (30.0%)	10 (47.6%)	0.2
Vomiting	13 (28.3%)	5 (14.7%)	0.2	5 (16.7%)	0 (0%)	0.049; OШ нeт
Photophobia	15 (32.6%)	5 (14.7%)	0.07	9 (30.0%)	1 (4.8%)	**0.03;** **8.0;** **1.0–33.7**
Phonophobia	11 (23.9%)	2 (5.9%)	**0.03;** **5.0;** **1.0–24.4**	7 (23.3%)	1 (4.8%)	0.07
Aura	1 (2.2%)	1 (2.9%)	0.8	0 (0%)	0 (0%)	-
** Location**						
Frontal	16 (34.8%)	10 (29.4%)	0.6	11 (36.7%)	8 (38.1%)	0.9
Temporal	21 (45.7%)	15 (44.1%)	0.9	16 (53.3%)	9 (42.9%)	0.5
Frontotemporal	12 (26.1%)	15 (44.1%)	0.09	9 (30.0%)	7 (33.3%)	0.8
Occipital	18 (39.1%)	9 (26.5%)	0.2	17 (56.7%)	4 (19.0%)	**0.007;** **4.4;** **1.3–14.6**
Parietal	16 (34.8%)	3 (8.8%)	**0.007;** **5.5;** **1.5–20.9**	7 (23.3%)	1 (4.8%)	0.07; 5.9; 0.7–50.6
Hemicrania	10 (21.7%)	2 (5.9%)	0.05; 4.4; 0.9–21.8	3 (10.0%)	1 (4.8%)	0.5
** Side**						
Unilateral	23 (50.0%)	5 (14.7%)	**0.001;** **5.8;** **1.9–17.6**	6 (20.0%)	4 (19.0%)	0.9
Right	14 (30.4%)	3 (8.8%)	**0.02;** **4.5;** **1.2–17.3**	2 (6.7%)	2 (9.5%)	0.7
Left	9 (19.6%)	2 (5.9%)	0.08	4 (13.3%)	2 (9.5%)	0.7
Bilateral	22 (47.8%)	27 (79.4%)	0.004; 0.2; 0.1–0.7	24 (80.0%)	16 (76.2%)	0.7
Alternating from one attack to the next	1 (2.2%)	2 (5.9%)	0.4	0 (0%)	1 (4.8%)	0.2
Simple analgesics ≥ 15 days per month	n/a	8 (23.5%)		2 (6.7%)	3 (14.3%)	0.4
Combine analgesics ≥ 10 days per month	n/a	4 (11.8%)		1 (3.3%)	5 (23.8%)	0.03; 0.1; 0.01–1.2
Triptans ≥ 10 days per month	n/a	0 (0%)		1 (3.3%)	0 (0%)	0.4

^a^ *n/a* Not acceptable

We compared changes in headache frequency during the 3 months observed period. The majority of patients had a gradually decreasing frequency of attacks (Table 2). However, 26% of the patients with persistent new type of headache and 38% of the patients with persistent headache with altered characteristics developed chronic headache ≥ 15 days per month.

Most patients with persistent headaches (39 patients, 70.9%) used analgesics for pain relief, and only a few (3 patients, 5.4%) used triptans. 35% of patients with persistent new type of headache and 38% of patients with persistent headache with altered characteristics used simple analgesics ≥ 15 days per month or combined analgesics ≥ 10 days per month and thus had medication overused headache. The use of these analgesics become 2.6 times more in three months in patients with persistent headache with altered characteristics. However, almost nobody used triptans ≥ 10 per month. Compared to previously, triptan use decreased significantly: 12 patients used them initially and only 3 patients used them at 3 months. Thus, most patients found triptans ineffective (Table 3) even if their headaches had migraine characteristics. Medication overuse headache developed in 17 of 55 patients (30.9%), among them 9 patients (16.4%) with persistent new type of headache and 8 (14.5%) patients with persistent headache with altered characteristics.

**Table 3:** Medical drugs for pain relief and their effect in patients with headache in three months after first-ever ischemic stroke compared to acute treatment of headache at onset of stroke

**Acute treatment**	**New type of headache**		**P, OR, 95% CI**	**Headache with altered characteristics**		**P, OR, 95% CI**
	**Headache at the onset of stroke (*n* = 46)**	**Headache in three months after stroke (*n* = 34)**		**Headache at the onset of stroke (*n* = 30)**	**Headache in three months after stroke (*n* = 21)**	
Simple analgesics < 15 days per month	5 (10.9%)	3 (8.8%)	0.8	7 (23.3%)	6 (28.6%)	0.7
Simple analgesics ≥ 15 days per month	n/a	8 (23.5%)	-	2 (6.7%)	3 (14.3%)	0.4
Effect of simple analgesics						
No effect	2 (4.3%)	3 (8.8%)	0.4	4 (13.3%)	5 (23.8%)	0.3
Minor effect	2 (4.3%)	4 (11.8%)	0.2	2 (6.7%)	1 (4.8%)	0.8
Medium effect	1 (2.2%)	3 (8.8%)	0.2	2 (6.7%)	2 (9.5%)	0.7
Complete effect	0 (0%)	1 (2.9%)	0.2	1 (3.3%)	1 (4.8%)	0.8
Combine analgesics < 10 days per month	10 (21.7%)	6 (17.6%)	0.7	8 (26.7%)	6 (28.6%)	0.9
Combine analgesics analgesics ≥ 10 days per month	n/a	4 (11.8%)	-	1 (3.3%)	5 (23.8%)	0.03; 0.11; 0.1–1.0
Effect of combined analgesics						
No effect	2 (4.3%)	2 (5.9%)	0.8	3 (10.0%)	3 (14.3%)	0.6
Minor effect	3 (6.5%)	3 (8.8%)	0.7	3 (10.0%)	5 (23.8%)	0.2
Medium effect	4 (6.2%)	4 (11.8%)	0.7	2 (6.7%)	2 (9.5%)	0.7
Complete effect	1 (2.2%)	1 (2.9%)	0.8	1 (3.3%)	1 (4.8%)	0.8
Triptans at the day of stroke onset < 10 days per month	2 (4.3%)	1 (2.9%)	0.7	10 (33.3%)	3 (14.3%)	0.1
Triptans ≥ 10 days per month	n/a	0 (0%)	-	1 (3.3%)	0 (0%)	0.2
Effect of triptans						
No effect	1 (2.2%)	0 (0%)	0.4	1 (3.3%)	2 (9.5%)	0.4
Minor effect	1 (2.2%)	1 (2.9%)	0.8	2 (6.7%)	1 (4.8%)	0.8
Medium effect	0 (0%)	0 (0%)	-	3 (10.0%)	0 (0%)	0.1
Complete effect	0 (0%)	0 (0%)	-	5 (16.7%)	0 (0%)	0.49

The following factors were associated with persistent headache after first-ever ischemic stroke (Table 4): lack of sleep (29.1%, *p* = 0.009; OR 2.3; 95% CI 1.2–4.3), localization of infarct in cerebellum (18.2%, *p* = 0.003; OR 3.0; 95% CI 1.4–6.6), stroke of undetermined etiology (50.9%, *p* = 0.003; OR 2.3; 95% CI 1.3–4.1), less than 8 points by NIHSS score (90.9%, *p* = 0.007; OR 3.4; 95% 1.4–8.6) and low prevalence of large-artery atherosclerosis (12.7%, *p* = 0.006; OR 0.3; 95 CI 0.2–0.80.

**Table 4:** Factors associated with persistent headaches after first-ever ischemic stroke

Associated factors (predictors)	Patients with persistent headache (*n* = 55)	Patients without headache (*n* = 468)	P, OR, 95% CI	Logistic Regression Analysis (exponentiated coefficients; 95% confidence intervals)
Mean age	60.9	63.5	0.2	
< 45 years old	2 (3.6%)	19 (4,1%)	0.9	.201* [.0309,1.31]
≥ 45 years old	53 (96.4%)	449 (95,9%)	0.9	
Females, n (%)	28 (50.9%)	210 (44.9%)	0.4	.89 [.381,2.08]
Smokers, n (%)	20 (36.4%)	201 (42.9%)	0.4	.608 [.265,1.39]
History of alcohol consumption				
Low-alcohol beverage, n (%)	11 (20.0%)	63 (13.5%)	0.2	1.7 [.586,4.9]
High-alcohol beverage, n (%)	8 (14.5%)	105 (22.4%)	0.2	.762 [.297,1.96]
History of diabetes, n (%)	13 (23.6%)	71 (15.2%)	0.1	6.81*** [2.13,21.7]
History of atrial fibrillation, n (%)	5 (9.1%)	74 (15.8%)	0.2	
** Lack of sleep, n (%)**	**16 (29.1%)**	**71 (15.2%)**	**0.009; 2.3; 1.2–4.3**	2.8*** [1.3,6.02]
Body mass index > 25, n (%)	35 (63.6%)	320 (68.4%)	0.5	.593 [.293,1.2]
Hyperglycemia, n (%)	23 (41.8%)	177 (37.8%)	0.6	1.04 [.46,2.35]
Hypercholesterinemia, n (%)	27 (49.1%)	193 (41.2%)	0.3	1.49 [.757,2.92]
Angina, n (%)	1 (1.8%)	131 (28.0%)	< 0.001; 0.05; 0.01–0.3	.379* [.136,1.06]
Myocardial infarction, n (%)	4 (7.3%)	47 (10.0%)	0.5	3.99** [1.18,13.4]
History of hypertension, n (%)	50 (90.9%)	441 (94.2%)	0.3	.619 [.188,2.04]
Stroke in first-degree relatives, n (%)	10 (18.2%)	148 (31.6%)	0.04; 0.5; 0.3–0.9	.857 [.413,1.78]
**History of previous headaches**				
Migraine, n (%)	5 (9.1%)	68 (14.5%)	0.3	27** [1.73,423]
** Tension-type headache, n (%)**	**16 (29.1%)**	**286 (61.1%)**	** < 0.001; 0.3; 0.1–0.5**	.0538** [.00379,.764]
TOAST classification				
** Large-artery atherosclerosis, n (%)**	**7 (12.7%)**	**143 (30.5%)**	**0.006; 0.3; 0.2–0.8**	.638 [.175,2.33]
Small-vessel occlusion (lacune), n (%)	7 (12.7%)	107 (22.8%)	0,09	.172** [.0451,.66]
Cardioembolism, n (%)	4 (7.3%)	63 (13.5%)	0.2	.378 [.0896,1.6]
** Stroke of undetermined aetiology, n (%)**	**28 (50.9%)**	**144 (30,8%)**	**0.003; 2.3; 1.3–4.1**	1.87 [.743,4.7]
Other etiologies, n (%)	9 (16.4%)	11 (2.35%)	** < 0.001;8.1; 3.2–20.7**	
Peripheral artery disease, n (%)	3 (5.5%)	11 (2.4%)	0.2	3.68 [.692,19.6]
Posterior circulation stroke, n (%)	10 (18.2%)	72 (15.4%)	0.6	.382 [.0912,1.6]
Localization of infarct				
Frontal, n (%)	8 (14.5%)	85 (18.2%)	0.5	1.1 [.379,3.18]
Parietal, n (%)	7 (12.7%)	82 (17.5%)	0.4	.874 [.315,2.43]
Temporal, n (%)	6 (10.9%)	53 (11.3%)	0.9	.749 [.242,2.32]
Occipital, n (%)	6 (10.9%)	48 (10.3%)	0.9	1.25 [.414,3.79]
Subcortical, n (%)	13 (23.6%)	148 (31.6%)	0.5	1.4 [.55,3.59]
** Cerebellum, n (%)**	**10 (18.2%)**	**32 (6.8%)**	**0.003; 3.0;** **1.4–6.6**	7.07*** [1.81,27.6]
The brain stem, n (%)	5 (9.1%)	52 (11.1%)	0.6	1.63 [.502,5.32]
Size of infarct				
3–15 mm, n (%)	9 (16.4%)	151 (32.3%)	0.02; 0.4; 0.2–0.9	.63 [.191,2.08]
** > 15 mm, n (%)**	**27 (49.1%)**	**195 (41.7%)**	**0.3**	.902 [.339,2.4]
NIHSS score				
** Less than 8 points, n (%)**	**50 (90.9%)**	**349 (74.6%)**	**0.007; 3.4;** **1.4–8.6**	4.21*** [1.52,11.7
8 – 16 points, n (%)	5 (9.1%)	118 (25.2%)	0.008; 0.3; 0.1–0.8	
More than 16 points, n (%)	0 (0%)	1 ( 0.2%)	0.7	

*Abbreviation*: *NIHSS* National Institutes of Health Stroke Scale l, *CI* Confidence interval, *OR* Odds ratio

^*^ *p* < 0.1

^**^ *p* < 0.05

^***^ *p* < 0.01

## Discussion

The main findings of our study were: 1) 10.4% of patients had persistent headaches attributed to stroke, most had a new type of headache; 2) migraine-like headaches and tension-type-like headaches were equally common; 3) persistent headache after stroke is characterized by gradually decreased in attack frequency and accompanying symptoms (photo-, phonophobia, vomiting), unilateral headache changed into bilateral; 4) medication (analgesics) overuse headache developed in 31% of patients; 6) lack of effect of triptans in almost half of the patients with migraine-like persistent headache; 7) persistent headache was associated with infarct in the cerebellum, good neurological status at stroke onset, strokes of undetermined aetiology, low prevalence of large-artery atherosclerosis and lack of sleep.

The occurrence of persistent headaches in our study was 10.4%, which is in accordance with previous studies [5, 6]. However, only 1% of persistent headaches after stroke was found in a study which included persistent headaches at the chronic stage of ischemic stroke, average follow-up time was 15.6 months [7].

As distinct from other previous studies [5–8], we found an equal number of migraine-like (25/55) and tension-type-like headaches (25/55) in patients with persistent headaches. Other studies showed a higher prevalence of tension-type headaches [5–8]. One study demonstrated changes in characteristics of headaches from migraine-type to tension-type in almost half of stroke survivors at the chronic stage of ischemic stroke [7]. The authors of this study suspected that stroke had a modulating effect on the headache in most stroke survivors. Another study reported that persistent headaches were more likely to be probable tension-type (50%) than probable migraine (31%) [6].

Transformation of unilateral headache into bilateral and decreasing frequency of attacks and accompanying symptoms are interesting observations of our study. We can explain this by chronification of headaches and development of medication overuse headache in almost half of patients with migraine-like headaches which was not described before. It was possible to detect this transformation thanks to the inclusion of patients with first-ever ischemic stroke and a clear description of headache characteristics at stroke onset. Other studies included patients with prior strokes which can produce persistent headaches and these headaches can interfere with the current headache caused by recurrent stroke [6, 7].

Interestingly, patients with persistent headaches were significantly more likely to use analgesics than patients with acute headaches attributed to ischemic stroke. Medication overuse headache developed in 31% of patients in 3 months after stroke. We can explain this by the preserved severe intensity of persistent headache in most of the patients and the development of chronic headache ≥ 15 days per month in ≈30% of the patients. The Hansen study reported that persistent post-stroke headaches may be more frequent and severe than headaches associated with acute stroke [6]. Some studies found that among patients with post-stroke pain syndromes patients with headaches used analgesics significantly more frequently than patients with post-stroke pain affecting a limb or another location (32% vs 17%) [9]. This phenomenon may also worsen persistent headaches after stroke.

The low effectiveness of triptans for pain relief is a very interesting observation which was not described before. Although almost half of the patients had a migraine-like persistent headache after stroke, triptans were ineffective. Probably the pathophysiology of persistent headaches and spontaneous migraine is different. This may be found in future for other migraine-like secondary headaches. It is necessary to take into account that triptans should not be used to treat acute migraine or migraine-like attacks in patients with uncontrolled hypertension, specifically during hypertensive crisis [10]. This situation can arise in patients with persistent poststroke headache.

The majority of persistent headaches were due to infarcts in the cerebellum (*p* = 0.003; OR = 3.0; 95% CI 1.4–6.6). Beyond this, no relation between side/localization of headache and side/localization of infarct was apparent. This is in accordance with other previous studies [5, 6]. It can be explained by the difference in the trigeminal and autonomic innervation of the meninges overlying the posterior cerebral cortices and cerebellum which increases the possibility that infarcts in these areas may have a greater ability to stimulate pain-sensing trigeminal and cervical dorsal root ganglion afferent fibres innervating the coverings overlying these regions [5]. Our previous study found an association between headaches attributed to stroke and infarcts of the cerebellum (*p* = 0.02, OR = 2.3, 95% CI = 1.1–4.8) [3].

Strokes of undetermined aetiology prevailed in patients with persistent headaches after stroke despite our previous study showing that headaches attributed to acute ischemic stroke were associated with cardioembolic stroke [3]. We can assume that paroxysmal atrial fibrillation was undetected during daily monitoring of heart rhythm in many patients and therefore in the absence of other courses of stroke these strokes were defined as undetermined aetiology. If more prolonged (for example, 7 days) EKG monitoring had been possible, the percentage of cardioembolic stroke could have been higher.

As district from previous studies [5, 8] we did not find a high prevalence of previous headaches (migraine or tension-type headache), female sex and people < 45 years old among patients with persistent headaches after stroke. It can be explained by carefully distinguishing between current and previous headaches according to the main principles of ICHD 3 and including only patients with first-ever ischemic stroke.

Our study confirmed that good neurological status at onset of stroke is a predictor not only for acute headache attributed to ischemic stroke (*p* = 0.01, OR = 2.5, 95% CI = 1.2–4.9) but also for persistent headache after stroke (*p* = 0.007; OR 3.4; 95% 1.4–8.6). We can explain this by preserving those brain regions implicated in neural networks responsible for pain processing and good arterial elasticity of cerebral arteries without severe atherosclerosis.

Lack of sleep was also associated with acute headache attributed to ischemic stroke (*p* = 0.002, OR 2.3, 95% CI 1.4–4.0) and persistent headache after stroke (*p* = 0.009; OR 2.3; 95% CI 1.2–4.3). We assume that lack of sleep can provoke increased pain sensitivity. Although we did not examine in this study the presence in our patients of obstructive sleep apnea, post-stroke depression, post-stroke generalized anxiety and fatigue, these disorders also have been linked to post-stroke headache and pain [8].

Disability induced by the sequelae of the ischaemic stroke must always be taken into account by incision of persistent poststroke headache and the problems of pain control in patients who have an obvious polypharmacy situation and use many drugs for secondary stroke prevention. It is necessary to remember that polypharmacy exposes patients with comorbidities (particularly elderly patients) to an increased risk of drug-specific adverse events and drug–drug interactions [11].

### Strengths and weaknesses of the present study

The present study included prospective interviews of a large number of patients in the acute phase of first-ever ischemic stroke (*n* = 550), diagnostics of headaches attributed to acute ischemic stroke according to ICHD 3 (*n* = 76), follow-up of the patients with these headaches (*n* = 61) > 3 months and their recurrent interviews 3 months after a stroke about persistent headaches after stroke. Headache characteristics were recorded at the onset of ischemic stroke and compared at various follow-up points to identify the group that truly has a persistent headache that occurred around the time of stroke. Clear inclusion/exclusion criteria were used in each stage of the study and standard diagnostic criteria of ICHD 3 were used for the diagnosis of persistent headache attributed to ischemic stroke. We reported descriptive characteristics of these headaches, including headache quality, severity, duration, accompanying symptoms, changes in their frequency each month after stroke for 3 months, medical drugs for pain relief and their effect in patients with a headache at the onset of stroke and in three months. Besides, we tried to analyze factors associated with the transformation of an acute headache to a persistent headache including ischemic stroke aetiology and location.

Our study did access for preexisting diagnoses of primary headache disorders before stroke which were defined using ICHD 3. All persistent headaches were compared with headaches at onset of ischemic stroke which were accurately distinct from the previous headaches, we distinguished headaches attributed to ischemic stroke and usual headaches. This led to the prevention of misclassification bias. A neurologist who interviewed patients 3 months after stroke did not evaluate the patients previously and was blinded to the presence of headache at stroke onset. Recall bias was prevented by using of prospective design of the study and inclusion of patients without memory problems who can give clear descriptions of present and previous headaches. However, the patients with severe stroke, impaired consciousness and aphasia were excluded, this can predispose to selection bias possibly contributing to underestimation. Besides, all patients were affiliated only with one city hospital, this decreased generalizability of our results.

## Conclusion

Persistent headache attributed to ischemic stroke is not rare and frequently leads to medication overuse. The problem is often neglected because of other serious consequences of stroke but actually, it has a considerable impact on quality of life. It should be a focus of interest in the follow-up of stroke patients.

## Data Availability

The datasets used and analyzed during the current study are available from the corresponding author on reasonable request.

## Abbreviations

CT: Computed Tomography
MRI: Magnetic resonance imaging
DWI: Diffusion-weighted imaging
TIA: Transient ischemic attacks
OR: Odds ratio
CI: Confidence interval
ICHD-3: International Classification of Headache Disorders
